# Role of Folate in Liver Diseases

**DOI:** 10.3390/nu16121872

**Published:** 2024-06-14

**Authors:** Minlan Yang, Dingye Wang, Xiyuan Wang, Jie Mei, Quan Gong

**Affiliations:** School of Medicine, Yangtze University, Jingzhou 434020, China

**Keywords:** folate, folate metabolism, NAFLD, ALD, liver fibrosis, HCC

## Abstract

Folate is a water-soluble B vitamin involved in the synthesis of purines and pyrimidines and is one of the essential vitamins for human growth and reproduction. Folate deficiency due to low dietary intake, poor absorption of folate, and alterations in folate metabolism due to genetic defects or drug interactions significantly increases the risk of diseases such as neural tube defects, cardiovascular disease, cancer, and cognitive dysfunction. Recent studies have shown that folate deficiency can cause hyperhomocysteinemia, which increases the risk of hypertension and cardiovascular disease, and that high homocysteine levels are an independent risk factor for liver fibrosis and cirrhosis. In addition, folate deficiency results in increased secretion of pro-inflammatory factors and impaired lipid metabolism in the liver, leading to lipid accumulation in hepatocytes and fibrosis. There is substantial evidence that folate deficiency contributes to the development and progression of a variety of liver diseases, including non-alcoholic fatty liver disease (NAFLD), non-alcoholic steatohepatitis (NASH), alcoholic liver disease (ALD), viral hepatitis, hepatic fibrosis, and liver cancer. Here we review key studies on the role of folate in the pathophysiology of liver diseases, summarize the current status of studies on folate in the treatment of liver diseases, and speculate that folate may be a potential therapeutic target for liver diseases.

## 1. Introduction

Folate, which belongs to the vitamin B9 family, was first purified and termed folate by Lucy Wills in 1931 [1]. Man-made pure crystalline forms of folate (i.e., folic acid) were first manufactured in 1943 [2]. Folate is involved in the synthesis of nucleic acids and proteins and is an essential vitamin for growth and reproduction. The active intermediate of folate, 5,10-methylenetetrahydrofolate (5,10-MTHF), converts deoxyribonucleotides (dUMPs) to deoxythymidine nucleotides (dTMPs), which are the structural units that make up DNA molecules [3]. Therefore, folate is required for both the replication of DNA during cell division and the repair process after DNA damage in the nucleus. In addition, as a carrier of one-carbon (1C) units, folate facilitates the transport of 1C units that are closely related to important physiological processes, including purine and thymidine synthesis, amino acid homeostasis, epigenetic maintenance, and redox defense. Tetrahydrofolate (THF) is the main form of folate in organisms, and 1C units participate in biosynthesis and metabolism when THF is used as a carrier. When folate deficiency blocks the delivery of 1C units, nucleic acid synthesis and amino acid metabolism are affected [4]. Nucleic acid and protein synthesis is the material basis for cell proliferation, growth, and development. Therefore, folate plays an extremely important role in cell division and tissue growth in organisms. In addition, folate is related to the production of methyl donors that are required for epigenetic regulation, such as DNA methylation. In turn, DNA methylation is involved in the regulation of chromatin dynamics and DNA accessibility and can prevent the binding of certain transcription factors to DNA motifs or recruit proteins with methyl-binding structural domains that regulate transcription initiation [5]. 5-methyltetrahydrofolate (5-MTHF) is the active form of folate, and absorbed folate is metabolized to 5-MTHF in the intestine or liver. Folate is usually first reduced to dihydrofolate (DHF) by dihydrofolate reductase (DHFR) and further converted to tetrahydrofolate (THF) for entry into the folate pool. THF is dependent on the serine hydroxymethyltransferase (SHMT) enzyme of vitamin B6 for its conversion to 5, 10-MTHF, which is then irreversibly converted to 5-MTHF by methylenetetrahydrofolate reductase (MTHFR). This reaction process produces mobile methyl groups that allow homocysteine (Hcy) to synthesize methionine in the presence of vitamin B12-dependent methionine synthase. Methionine is the substrate for S-adenosylmethionine (SAM). In turn, SAM is a methyl donor for several methylation reactions. Low levels of folate reduce genomic DNA methylation [6]. Thus, folate participates in epigenetic modifications through methylation and other mechanisms to maintain normal body homeostasis. Hcy is the product of methionine demethylation. Hyperhomocysteinemia (HHcy) can be induced by folate deficiency [7]. An increase in Hcy can produce a large number of oxidizing free radicals, causing damage to vascular endothelial cells and lipid peroxidation, thus causing damage and destruction of blood vessels. Dietary supplementation with folate can reduce the level of Hcy, affect endothelial nitric oxide synthase (eNOS) in vascular endothelial cells and vascular oxidative stress, and reduce the risk of cardiovascular diseases. In addition, folate is necessary for the formation of hemoglobin in red blood cells. Insufficient intake of folate impairs the maturation of red blood cells, leading to macrocytic anemia and leukopenia [8]. Folate deficiency can also affect skeletal muscle development, leading to muscle weakness and difficulty walking, and some studies have shown that folate has a positive effect on skeletal muscle cell development [9]. Therefore, folate is necessary for a variety of organisms and microorganisms. The liver is the primary organ responsible for the storage and metabolism of folate. There is increasing evidence that folate deficiency or abnormal folate metabolism contributes to the occurrence and progression of various liver diseases and plays an important role in the pathogenesis of these diseases. Therefore, a better understanding of the role of folate in the development of these diseases may help identify new treatment strategies. This review discusses some of the roles of folate and folate metabolism in the pathophysiological progression of liver diseases.

## 2. Folate Metabolism

**Figure 1:**
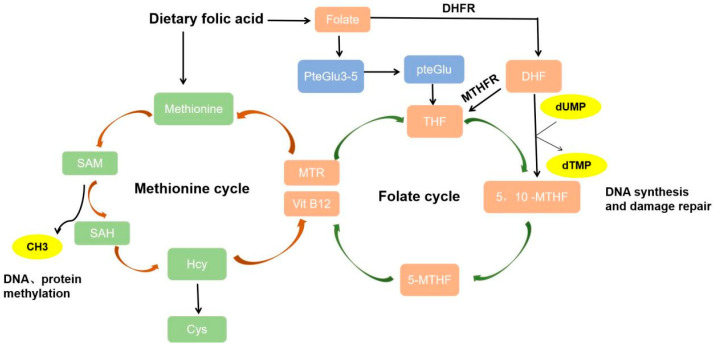
The process of dietary folic acid metabolism. After dietary folic acid is absorbed, it is reduced to DHF by DHFR and regenerated into THF by MTHFR. Meanwhile, the polyglutamylated folates PteGlu3–5 are decomposed into monoglutamate folate PteGlu, and transformed into THF. In the folate cycle, a released 1C unit from serine or glycine is transferred to THF, forming 5,10-MTHF. 5,10-MTHF is reduced to the active 5-MTHF under the action of MTHFR. THF carries 1C units to form 5,10-MTHF and 5-MTHF. 5,10-MTHF converts dUMP to dTMP, which is necessary for DNA synthesis and repair. In the methionine cycle, methionine is catalyzed to form SAM, SAM is demethylated to produce SAH, which is deadenosined to form Hcy. Released CH3 is the main source of methylation of DNA and protein. Methionine can be regenerated from Hcy in the presence of MTR and VitB12.

Polyglutamylated folate is the predominant form of folate in all organisms, and polyglutamylated folate must be converted into monoglutamate folate by γ-glutamyl hydrolases, which are secreted by the mucosal cells of the small intestine before it can be absorbed by the liver and small intestine. Synthetic folic acid can be directly absorbed, and the absorption rate is greater. Folate metabolism mainly includes three physiological processes, as shown in Figure 1.

### 2.1. Folate Metabolic Process

After folate is absorbed, it is reduced to the intermediate product DHF by DHFR and regenerated into THF. The 1C unit from serine or glycine is transferred to THF, forming 5,10-MTHF. 5,10-MTHF is reduced to the active 5-MTHF under the action of MTHFR. 5-MTHF is the main form of folate in the blood.

### 2.2. Folate Mediates One-Carbon Metabolism and Promotes DNA Synthesis

Folate is reduced to DHF by DHFR and then converted to THF during intestinal absorption and tissue transport. THF is a carrier of 1C units and transfers 1C units. THF carries these carbon units to form 10-formyltetrahydrofolate (10-formyl-THF), 5,10-MTHF, and 5-MTHF. 5,10-MTHF converts dUMP to dTMP, and dUMP is phosphorylated to deoxyriboside triphosphate by the addition of methyl groups. Thymidine triphosphate (TTP), also called deoxythymidine triphosphate (dTTP), is one of the four deoxyribonucleic acids necessary for DNA synthesis and repair.

### 2.3. Folate Mediates the Methionine Cycle

During the dietary intake of methionine or methionine amino acids, methionine is catalyzed by adenosyltransferase and reacts with ATP to form SAM, the methyl group in SAM becomes the active methyl group, and SAM is called the active methionine. SAM is catalyzed by methyltransferases, which transfer methyl groups to other substances, such as DNA. SAM is demethylated to produce S-adenosine homocysteine (SAH), which leads to the production of Hcy. Hcy then accepts the methyl group on 5-MTHF and regenerates methionine, thus forming a cycle known as the methionine cycle. 

In general, as a coenzyme in the 1C unit transferase, folate acts as a 1C unit transmitter. Folate metabolism provides materials for the synthesis of purine and thymine and participates in the synthesis of DNA and RNA. In addition, folate mediates the metabolic process of amino acids and promotes the mutual conversion of Hcy and methionine. Finally, folate can provide SAM. SAM is a methyl donor that transfers methyl groups to specific bases through covalent bonding under the action of DNA methyltransferases (DNMTs), which leads to DNA methylation modification. 

## 3. Folate and Liver Diseases

**Figure 2:**
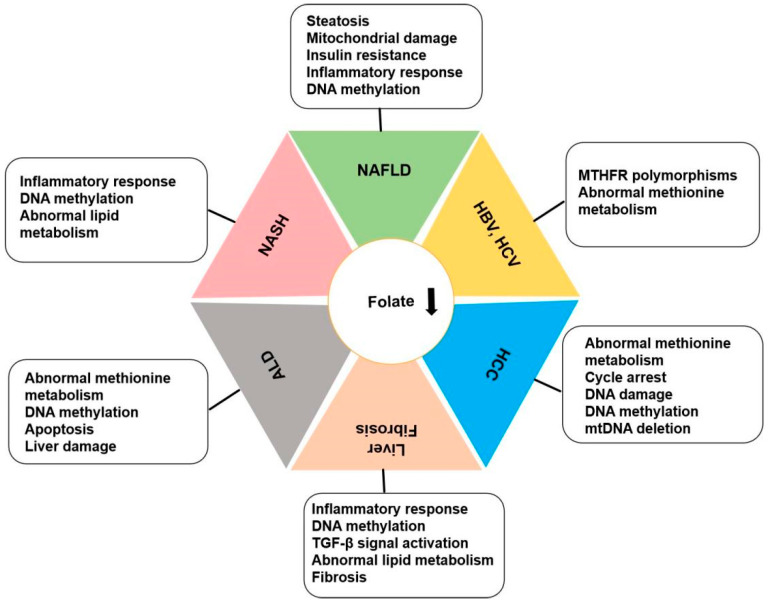
Mechanisms and potential therapeutic targets for folate in liver diseases.

The liver serves as the primary storage site for folate and plays a crucial role in lipid synthesis, indicating a potential impact of folate levels on liver metabolism. Numerous animal studies have demonstrated that folate deficiency can result in disruptions in protein regulation and aberrant gene expression in the liver, leading to elevated triglyceride levels and reduced plasma high-density lipoprotein (HDL) concentrations. Folate insufficiency is implicated in the pathogenesis of liver diseases through its influence on methionine metabolism, DNA synthesis and stability, and epigenetic modulation of gene expression [10]. Research findings indicate a progressive decrease in serum folate levels as hepatitis C virus infection advances from chronic hepatitis to cirrhosis to liver cancer [11]. Folate deficiency leads to increased secretion of pro-inflammatory factors in the liver, impairs lipid metabolism, and leads to excess fat accumulation in hepatocytes and fibrosis [12,13]. This evidence suggests that folate may play a role in the onset and development of liver diseases. In addition, in vivo studies have confirmed that folate deficiency affects liver homeostasis in offspring. Folate regulates liver fatty acid metabolism. In the livers of female mice, a high-folate/high-vitamin B12 diet reduces total fatty acid and desaturase activity. Low prenatal folate and vitamin B12 levels have significant effects on the regulation of genes and enzymes related to lipid metabolism in the liver of adult female mice and their offspring [14]. Both maternal and paternal folate deficiency during mating affect the folate content and DNA methylation status in the liver of rats after birth, thus affecting the liver function of offspring [15]. Decreased level of serum folate is closely related to abnormal liver function and the occurrence and development of liver diseases, as shown in Figure 2. 

### 3.1. Folate and NAFLD, NASH

Nonalcoholic fatty liver disease (NAFLD) is characterized by the excessive deposition of lipids in hepatocytes in addition to alcohol and other specific liver-damaging factors. Nonalcoholic steatohepatitis (NASH) is a distinct, inflammatory subtype of NAFLD, accompanied by hepatic steatosis and evidence of hepatocyte injury and inflammation, with or without liver fibrosis. Substantial evidence suggests that folate deficiency contributes to the development of NAFLD. Low serum folate levels are associated with the histological severity of NASH [16]. One cross-sectional study that included data from 5417 patients from the 2011-2018 National Health and Nutrition Examination Survey (NHANES), and investigated the relationships between serum folate and NAFLD and advanced fibrosis (AHF). Studies have shown that higher serum folate levels are associated with a lower incidence of NAFLD in U.S. adults [17]. These findings indicate that patients with NAFLD may need appropriate folate supplementation. Compared with non-NAFLD patients, patients with NAFLD had significantly greater red blood cell folate levels and lower serum vitamin B12 and folate levels. This also means that people with NAFLD may need appropriate folate supplementation. Compared with non-NAFLD patients, patients with NAFLD had significantly greater red blood cell folate levels and lower serum vitamin B12 and folate levels. Erythrocyte folate has been independently associated with an increased risk of NAFLD [18]. By measuring the serum folate concentration and lipid content of NAFLD patients at different times, and conducting regression analysis to establish a linear mixing model, researchers found that an increase in serum folate levels can significantly reduce the expression of genes involved in fatty acid synthesis, while the expression of genes involved in fatty acid oxidation is increased. An increase in folate concentration may lead to a dramatic reduction in lipid accumulation in the liver [19]. These findings suggest that folate deficiency may promote the development of liver diseases, and monitoring serum folate levels has diagnostic and therapeutic significance for the management and follow-up of NAFLD patients.

#### 3.1.1. Folate Mediates One-Carbon Metabolism to Regulate NAFLD Progression

1C units refer to units containing a carbon atom, including methyl, methylenyl, and formyl groups. The process of 1C metabolism is the process of the generation and transfer of 1C units in the biological process. The 1C metabolic pathway can be combined with the folate and methionine cycles, contributing to nucleotide synthesis, lipid metabolism, and maintaining cellular redox homeostasis. The liver is a major site of 1C metabolism and lipid metabolism, and these pathways interact. The most abundant phosphatidylcholine (PC) is synthesized by adding three methyl groups to phosphatidylethanolamine (PE) under the catalysis of phosphatidylethanolamine n-methyltransferase (PEMT). In addition, 1C metabolism affects cellular energy homeostasis and immune function, which in turn affects liver lipid metabolism and liver disease progression. Both genetic and dietary animal models have demonstrated that changes in 1C metabolism are closely related to lipid metabolism in the liver. High dietary fat can induce NAFLD and disrupt 1C metabolism. Low levels of endogenous folate in rodents disrupt folate-dependent 1C metabolism, and may be associated with the development of metabolic diseases such as NAFLD [20]. Methionine, serine, glycine, and choline are the main sources of 1C units. A deficiency of methionine or choline in the diet can lead to steatosis. The methionine and choline deficiency (MCD) diet model is the most common NAFLD model. However, the use of the MCD model remains somewhat controversial, as MCD induces significant weight loss in NAFLD/NASH mice and does not cause obesity or insulin resistance, which are common in NAFLD. In addition, some drugs that disrupt 1C metabolism, such as methotrexate, can induce fatty liver diseases and cause liver injury [21]. Recent evidence suggests that disruption of 1C metabolism impairs mitochondrial function by limiting thymine biosynthesis [22]. The 1C unit transfer mediated by folate through serine hydroxymethyltransferase (SHMT) is also essential for mitochondrial biological processes. Reduced mitochondrial content impairs nutrient oxidation and leads to lipid accumulation. Impaired mitochondrial function can also promote apoptosis and affect normal immunity. These findings all point to the importance of folate-mediated 1C metabolism in maintaining a healthy liver.

#### 3.1.2. Folate Mediates DNA Methylation to Regulate NAFLD Progression

DNA methylation refers to the transfer of SAM as a methyl donor to a specific base, such as cytosine, through covalent bonding under the action of DNMTs and catalyzes the replacement of cytosine-phosphate-guanine (CpG) by methyl groups. The dinucleotide cytosine ring on the CpG island generates 5-methylcytosine (5mC) when DNA methylation occurs. DNA methylation is closely related to gene expression and usually inhibits gene transcription. Cytosine methylation inhibits gene expression either by preventing transcription factor interactions or by recruiting DNA binding domains with affinity for 5mC that interact with histone-modifying enzymes to form dense structures, thereby affecting chromatin structure and cell/tissue specific gene expression. Five DNMT enzymes have been identified in mammals, namely DNMT1, DNMT2, DNMT3A, DNMT3B, and DNMT3L. Among them, DNMT1, DNMT3A, and DNMT3B are related to the activity of methyltransferase. The main function of DNMT1 is to maintain the DNA methylation pattern of existing CpG islands, which is highly expressed in mammals and is the most important enzyme involved in regulating DNA methylation. DNMT1 can methylate newly synthesized DNA chains under the guidance of methylated DNA templates, which is very important for the maintenance of DNA methylation patterns in genetic imprinting. DNMT3a and DNMT3b are involved in the de novo methylation of DNA, which is the conversion of unmethylated CpG to methylated CpG. To maintain a stable methylation state, DNMT3A and DNMT3B bind to DNMT1. Folate is a dietary methyl donor that produces SAM, a substrate for all methyltransferases involved in regulating gene expression in liver injury. Methylation and sulfur transfer pathways compete for Hcy formed from methionine. MTHF is used as a methyl donor to remethylate Hcy and forms the methionine cycle, which plays a role in protecting methionine. 

Hepatic insulin resistance and steatosis are major features of metabolic liver diseases, especially metabolic diseases such as NAFLD and type 2 diabetes (T2D). Epigenetic alterations, particularly DNA methylation, play a key role in the pathogenesis of insulin resistance. An increase in free fatty acids (FFAs) can promote mitochondrial translocation of the DNA methyltransferase DNMT1 by activating adenosine monophosphate (AMP)-activated protein kinase (AMPK), and further specifically induce transcription inhibition and mitochondrial oxidative phosphorylation of key genes that are related to respiratory chain complex I, which is encoded by mitochondrial DNA, ultimately leading to insulin resistance [23]. Folate is an important source of DNA methylation in 1C unit. A cross-sectional study from the NHANES of 1530 adults without diabetes revealed that there was a significant negative correlation between serum folate levels and insulin resistance [23]. These results suggest that folate may influence insulin resistance by mediating DNA methylation and lead to metabolic disorders in the liver. The intake of high amounts of folic acid leads to a deficiency of MTHFR, which leads to reduced methylation capacity and abnormal lipid metabolism in the liver [24]. On the other hand, folate supplementation in mice fed a high-fat diet reduced liver lipids and inhibited inflammatory responses [25]. Whole-genome DNA methylation analysis of peripheral white blood cells from patients with NAFLD revealed that CpGs in the promoters of PRKCE and SEC14L3 were hypomethylated and had high gene expression levels [26]. One study used data from the Neonatal Epigenetics Study (NEST) to analyze the relationship between differential DNA methylation and liver fat content (LFC), and liver damage in preadolescent children. LFC was found to be associated with 88 differentially methylated regions (MDRs) and 106 CpGs, of which two CpG loci, cg25474373 and cg07264203, located near the RFTN2 and PRICKLE2 genes, were significantly associated with the diagnosis of NAFLD [27]. There were 467 dinucleotides with abnormal methylation status in NASH patients, and eight genes related to metabolism and the onset of NAFLD, including GALNTL4, ACLY, IGFBP2, PLCG1, PRKCE, IGF1, IP6K3, and PC [28]. More than 1000 differentially methylated regions were also found in mouse models of NASH induced by a high-fructose/high-cholesterol diet, suggesting that DNA methylation is involved in regulating NASH development. The methylation levels of genes such as TGFB, MSN, IQGAP1, CYBA, and FCGR1 were significantly reduced, and increased expression may be the main cause of NASH [29]. This finding suggests that genomic DNA methylation may play an important role in both the onset and progression of NASH.

#### 3.1.3. The Role of Folate Receptors in NAFLD 

Mammals lack the ability to synthesize folate autonomously, so they must rely on specific transporter or receptor proteins to utilize external folate. Folate is transported across membranes in three ways, the first of which is to promote folate uptake from food through widely distributed reductive folate carriers. The second way is through proton-coupled folate transporters, which use membrane proton gradients to mediate folate transport into cells. The third mechanism is by transporting through folate receptors (FRs), which consist of four main glycopeptide molecules, FR-α, FR-β, FR-γ, and FR-δ. FR-α, also known as FOLR1 or folate-binding protein, has a high affinity for folate and is a glycoprotein anchored to the cell membrane by glycosylated phosphatidylinositol, transporting folate through receptor-mediated endocytosis. Folate binds to FRs and enters the cytoplasm through endocytosis, activates the cellular regulatory signaling network, and acts as a transcription factor. Resident macrophages activate the expression of folate receptor β (FR-β), and FR-β transcription levels are elevated in both NASH fat and non-fat samples [30], suggesting that FRs may be used for drugs that directly target the liver. Folate metabolism is involved in many physiological processes, and the most important factor in the pathogenesis of NAFLD is that folate mediates various mechanisms, and ultimately regulates the liver lipid synthesis and inflammatory processes.

### 3.2. Folate and Alcoholic Liver Disease

Alcoholic liver disease (ALD) is a liver disease caused by long-term heavy drinking. It usually begins with fatty liver disease, which can progress to alcoholic hepatitis, liver fibrosis and cirrhosis. The decrease in the serum folate level in ALD patients is mainly because drinking alcohol produces heat and reduces the food demand of the patients, resulting in reduced intake of VitB12 and folate. Long-term heavy drinking will damage the intestinal mucosa, resulting in poor absorption of folate and VitB12, and a high concentration of alcohol will directly damage the stomach, small intestine, and even the pancreas, resulting in reduced absorption of VitB12 and folate. Alcohol damage reduces the ability of the liver to store vitamin B12 and folate, and the ability to store and convert folate into THF.

#### 3.2.1. Folate Mediates Methionine Metabolism in ALD

Long-term alcohol exposure impairs folate absorption by inhibiting the expression of folate carriers and reducing the uptake of circulating folate by the liver and kidneys. Moreover, folate deficiency may increase changes in methionine metabolism in the liver of ALD patients and promote oxidative liver injury [31]. Folate deficiency is observed in most ALD patients. Both folate deficiency and alcoholism disrupt methionine metabolism in the liver. However, SAM prevents the development of ALD. In a micropig model of alcoholic liver injury, researchers observed steatohepatitis and abnormal methionine metabolism. These abnormalities include reduced levels of SAM and glutathione in the liver, as well as increased levels of lipid oxidation products. Methionine metabolism is regulated by folate, and folate deficiency and abnormal methionine metabolism are the main features of ALD [32]. Folate deficiency may contribute to the development of ALD by exacerbating abnormal methionine metabolism. Abnormal methionine metabolism is associated with DNA and lipid oxidation products and liver injury. Folate sufficiency prevents the early onset of methionine cycle-mediated ALD [33]. Decreased SAM also impairs nucleotide balance, leading to double-strand DNA breaks, oxidation, apoptosis, and increased risk of cancer [34].

#### 3.2.2. Folate Mediates DNA Methylation in ALD

Alcohol causes damage to cells and can alter the epigenetic states, including methylation and deacetylation of histones, hypermethylation of DNA, and changes in demethylation. Studies have reported a state of hypomethylation of certain genes in the liver after long-term exposure to alcohol, such as significantly reduced methylation of the MYC gene in ALD [35]. In pregnant rats subjected to long-term alcohol intake, the activity of DNMT in newborn mice was reduced and the state of hypomethylation was also observed. DNMT activity was also decreased in peripheral blood cells of ALD patients [36]. Folate is a dietary methyl donor that produces SAM, a substrate for all methyltransferases involved in regulating gene expression in liver injury. Long-term alcohol consumption also leads to a disturbance in the metabolism of 1C compounds, which ultimately leads to a decrease in the production of SAM, an important methyl donor in DNA and histone methylation [37]. In animals fed ethanol, long-term intake of ethanol decreases the ratio of SAM to SAH in hepatocytes and significantly impairs liver methylation. Folate deficiency accelerates the development of ALD while reducing SAM levels in the liver, resulting in abnormal gene expression and reduced production of the antioxidant glutathione. An increase in SAH levels and hypomethylation seriously affect the expression and activity of the apoptotic protease caspase-8, resulting in enhanced apoptosis of hepatocytes in ALD [38]. The combination of increased methylation requirements and long-term ethanol consumption leads to more pronounced liver damage [39].

In addition, folate supplementation can also improve mitochondrial function, inhibit ethanol-induced mitochondrial autophagy, and inhibit the release of mitochondrial cytochrome C into the cytoplasm, thus preventing hepatocyte apoptosis [40]. Folate supplementation can also reduce liver damage caused by alcohol, reduce oxidative stress, and restore the function of liver enzymes. ALD affects folate metabolism to cause folate deficiency in the body. Therefore, folate supplementation may have potential implications for alleviating ALD.

### 3.3. Folate and Liver Fibrosis and Cirrhosis

Liver fibrosis is the end result of a chronic inflammatory response and is characterized mainly by the deposition of type I collagen in the extracellular matrix (ECM), which destroys the normal physiological structure of the liver and leads to dysfunction. Pathologically, toxic, metabolic, or viral diseases can lead to liver injury and immune cell infiltration, thus activating liver stellate cells to transdifferentiate into collagen-producing myoblasts [41]. Activation of hepatic stellate cells (HSCs) is a prerequisite for liver fibrosis. Under normal circumstances, HSCs are quiescent. Once liver injury occurs, HSCs are activated and transformed into collagen secreting myofibroblasts, and then activated HSCs produce a large number of ECM components and pro-inflammatory mediators. Continuous injury disrupts the homeostasis between the secretion of collagen and the dissolution of collagen, and leads to the gradual accumulation of fiber scars to form progressive liver fibrosis. These fiber scars destroy the normal structure of the liver and affect its function.

#### 3.3.1. Folate Mediates DNA Methylation in Liver Fibrosis

DNA methylation is involved in the activation of HSCs, which promotes the progression of liver fibrosis. DNA methylation changes during hepatic fibrosis and HSC transdifferentiation, and DNA 5-mC and 5-hydroxymethylcytosine (5-hmC) methylation are key steps in HSC activation and fibrogenesis [41]. The DNA methylation inhibitor 5-aza-2′-deoxycytidine (5-AzadC) can inhibit the down-regulation of PTCH1 gene expression in activated HSCs. Abnormal methylation of the promoter of the PTCH1 gene further leads to the expression of the Gli1 and Smad3 genes associated with fibrosis in HSCs [42]. The DNA methylation status of the liver tissue of the rat model is changed, and some immune-related genes are hypermethylated. The activation of peroxisome proliferator-activated receptor γ (PPARγ) plays an important role in the occurrence and development of liver fibrosis. Some studies have shown that epigenetic modification of PPARγ is also involved in the regulation of liver fibrosis. Methylated CpG binding protein 2 (MeCP2) is involved in transcriptional regulation and mainly binds to DNA methylation sites to reverse transcriptional inhibition. Silencing MeCP2 or treatment with 5-AzadC inhibits the decrease in PPARγ expression and myofibroblast transformation in activated HSCs. In addition, down-regulation of MeCP2 can activate the NF-κB signaling pathway in the MFBs of myoblasts, thereby affecting the development of liver fibrosis [43]. In addition, DNA methylation modification of the transforming growth factor-β1 (TGF-β1) gene also plays an important role in the activation and proliferation of HSCs and the occurrence and development of liver fibrosis [44].

#### 3.3.2. Folate Receptors in Liver Fibrosis

FRs are involved in regulating liver fibrosis. TGF-β is a major pro-fibrotic cytokine that is closely related to fibrosis. Soluble folate receptor gamma (FOLR3) is a secreted protein that is elevated in the liver of patients with metabolic dysfunction-related steatohepatitis (MASH). Proteomic analysis has revealed that FOLR3 is the most significant MASH protein and is positively associated with increased fibrosis stage. Exogenous FOLR3 stimulates the production of ECM in HSCs, and TGF-β1 synergism further enhances this effect. FOLR3 interacts with the serine protease HTRA1 to activate TGF-β signaling [45].

#### 3.3.3. Folate Mediates 1C Metabolism in Liver Fibrosis

Sustained TGF-β1 signaling promotes fibrosis. In activated HSCs, folate is metabolically transferred to mitochondria, to maintain TGF-β1 signaling. Blocking mitochondrial folate-mediated 1C metabolism promotes the regression of liver fibrosis in NASH mice. Mitochondrial folate metabolism, ALA depletion, and TGF-βR1 production are feed-forward signals that maintain pro-fibrotic TGF-β1 signaling [45]. Targeting mitochondrial folate to mediate 1C metabolism is a potential strategy to promote the regression of liver fibrosis. In addition, folate deficiency impairs liver lipid metabolism, leading to increased secretion of liver pro-inflammatory factors, eventually leading to liver lipid accumulation and fibrosis. In American adults, higher serum folate levels are associated with a lower risk of developing advanced liver fibrosis (AHF) [17]. In preclinical models, dietary supplementation with vitamin B12 and folate may increase the expression of syntaxin17 (STX17) in the liver and restore its key role in autophagy, thereby slowing the progression of NASH and reversing the occurrence of inflammation and liver fibrosis [46].

### 3.4. Folate and Chronic Viral Hepatitis

With the progression of hepatitis C virus (HCV) infection, from chronic hepatitis to cirrhosis to hepatocellular carcinoma (HCC), serum folate levels gradually decrease [11]. HCV infection can directly or indirectly lead to hepatic steatosis. Folate deficiency indirectly leads to liver damage. Host genetic polymorphisms may also play a role in the development of steatosis. HCV infection significantly reduces the levels of vitamins B2, B6, and folate in red blood cells and/or plasma [47]. To mitigate other health risks, it is critical to monitor the nutritional status of patients infected with hepatitis B virus (HBV) or HCV and consider limiting or supplementing certain nutrients.

#### 3.4.1. Role of Folate Metabolism in Chronic Viral Hepatitis

Polymorphism of folate metabolism-related enzymes is closely related to enzyme function and the occurrence and development of liver diseases. MTHFR is the key enzyme involved in folate metabolism, and controls the nucleic acid synthesis and DNA methylation to regulate folate metabolism. MTHFR C677T mutations are associated with reduced enzyme activity. The MTHFR C677T polymorphism with TT genotype may be a factor in the increased risk of HBV-associated HCC in the Chinese population, especially in individuals whose HBV infection has persisted for more than 20 years [48]. The folate level is mainly affected by the MTHFR C677T polymorphism. The MTHFR C677T SNP may be associated with the development of early complications associated with HCV genotype 4 infection, such as dyslipidemia and decreased folate levels [49]. The polymorphisms of the MTHFR genes C677T (rs1801133, P.LU429VAL) and A1298C (rs1801131,P.Lu429ALA) decrease the enzyme activity. Polymorphisms of the MTHFR gene C677T and A1298C are associated with HBV infection in the Turkish population. The T allele of the C677T polymorphism is a risk factor for HBV infection. The CC-AA complex genotype has a protective effect on HBV infection, while the T-A haplotype is a risk factor for HBV infection [50].

#### 3.4.2. Role of Folate in Mediating Methionine Metabolism in Chronic Hepatitis

A course of disease lasting longer than 6 months is known as chronic liver disease (CLD), and common causes include hepatitis B and C viruses, NASH, ALD, and autoimmune liver disease. CLD is also an independent risk factor for HCC. The progression of CLD involves chronic substantive injury, sustained activation of the inflammatory response, and sustained activation of liver fibrosis and the wound healing response. During the occurrence and development of CLD, the changes in various components involved in methionine metabolism can affect the pathological status through multiple mechanisms. Diets lacking methionine are often used to establish models of CLD. The transformation of the methionine adenosine transferases MAT1A and MAT2A/MAT2B, key enzymes involved in methionine metabolism, is closely associated with fibrosis and HCC. Among them, MAT2A is responsible for SAM synthesis in extrahepatic normal tissue and cancer tissue, MAT1A is only responsible for SAM synthesis in normal liver tissue and bile duct epithelial cells, and MAT2B has no catalytic activity. SAM can balance the MAT1A/MAT2A ratio and prevent further progression of liver injury. SAM supplementation regulates antioxidant enzymes, synthesizes GSH, and alters the conversion of MAT1A/MAT2A. Moreover, one experiment also confirmed that interference with methionine adenosine transferase MAT1A and MAT2A or methionine metabolites can interfere with methionine metabolism and further reduce liver injury [51]. However, a randomized controlled trial revealed that SAM did not reduce liver damage in patients with HCV cirrhosis, possibly due to the high chemical reactivity and spontaneous breakdown of the methyl group of SAM, which can lead to adverse reactions. Therefore, intervention of methionine metabolism by targeting SAM for the treatment of CLD may require caution.

### 3.5. Folate and Liver Cancer: Hepatocellular Carcinoma

The risk factors for liver cancer mainly include chronic HBV or chronic HCV infection, aflatoxin contamination of food, long-term excessive drinking, smoking, and obesity, among others. With continuous research and exploration, nutritional factors and living environment are related to the occurrence and development of liver cancer, and alcoholism is the most important risk factor for liver cancer in North America and Northern Europe [52]. Increased intake of dairy products and high-sugar beverages is also associated with the development of liver cancer. Reduced dietary micronutrient intake has a significant impact on muscle health, and folate deficiency inhibits myoblast differentiation leading to sarcopenia. Sarcopenia is associated with poor outcomes in patients with cirrhosis and hepatocellular carcinoma [53]. Folate is an essential nutrient for cell growth and reproduction, and thus affects the growth of tumor cells and is closely related to liver cancer. 

#### 3.5.1. Folate Is Involved in the Regulation of HCC

Excessive drinking is a well-known risk factor for liver diseases and HCC. Alcohol consumption reduces folate absorption. In the absence of folate deficiency, the number of macrophages increases, sometimes accompanied by megaloblastic hyperplasia, suggesting that alcohol has a direct toxic effect on developing red blood cells [54]. Serum folate levels are inversely associated with the incidence of metabolic-associated fatty liver disease (MALFD) and metabolic dysfunction. Maintaining adequate serum vitamin C levels may help enhance the protective effect of folate against MALFD [55]. A cross-sectional study of 90 HCC patients analyzed the association between folate status and tumor progression in HCC patients. The serum folate level was negatively correlated with tumor size, tumor diversity, and metastasis. The serum folate level decreased gradually with increasing HCC progression. This suggests that low serum folate levels may be a risk factor for tumor progression [56]. The serum folate level of patients with primary liver cancer is significantly lower than that of the normal population [57]. Plasma folate levels are negatively correlated with HCC, with lower folate levels in patients with stage III, IV, or larger tumors [58]. A low serum folate concentration is independently associated with lower HCC survival [59]. High folate intake is inversely associated with liver injury and HCC. A study from the National Institutes of Health–AARP Diet and Health examined the effects of alcohol consumption and folate intake on the incidence and mortality of liver cancer. Increased folate intake may mitigate the effects of alcohol consumption on HCC development. Folate intake may help prevent alcohol-related HCC [60]. These findings suggest that folate supplementation may have a beneficial impact on the development of liver cancer.

However, taking too much folate may increase the risk of liver cancer. Excess Hcy can damage liver tissue structure, impair DNA repair processes, and increase the risk of liver cancer. Supplementation with excess folate can inhibit the degradation of MAT2A through the ubiquitin protein transferase VCIP135, indirectly accelerating the methionine cycle in cancer tissues, and promoting the progression of HCC [61]. The effect of dietary folic acid was evaluated in a rat HCC model, which revealed an increase in the number of cirrhosis cases and a decrease in the number of HCC cases following folate deficiency. This suggests that folate deficiency may help delay the progression of HCC. Excess folic acid is associated with an increase in the number of HCC patients and a decrease in the number of cirrhosis patients, suggesting that excessive intake of folic acid may promote the early progression of HCC [61]. A high-folate diet significantly promoted cancer development in DEN/high-fat diet-induced HCC mice [61]. In addition, studies have reported that folate deficiency in HCC leads to antioxidant stress and multidrug resistance, and folate supplementation improves the effectiveness of chemotherapy [62]. Folate has a dual role in the development of cancer, namely, by promoting or inhibiting the formation and progression of tumors. However, the underlying mechanism remains to be elucidated.

#### 3.5.2. Folate Mediates the Methionine Cycle in HCC

Folate metabolism combines with 1C metabolism and sulfur transfer pathways to regulate lipid and redox homeostasis. Adequate folate promotes Hcy metabolism and enhances the metabolic function of the liver, thereby protecting the liver from injury. Abnormal methionine metabolism is associated with the production of lipid oxidation products and liver injury. Folate deficiency may contribute to the development of liver disease by promoting abnormal methionine metabolism. Methionine metabolism is a key component of the 1C metabolism pathway. The disturbance of methionine metabolism can aggravate the damage caused by the pathological state of the disease. During the occurrence and development of CLD, the changes in various components involved in methionine metabolism affect the pathological state through various mechanisms. The key enzymes involved in methionine metabolism and transformation are closely related to liver fibrosis and HCC. Targeting related enzymes or downstream metabolites to interfere with methionine metabolism can reduce liver injury [61]. It is generally believed that methionine deficiency or blockade of methionine metabolism can inhibit the growth of tumor cells. Methionine deprivation or blockade of methionine catabolism decreases the proliferation of HCC cells [61]. In addition, a methionine-restricted diet improves the preclinical efficacy of chemotherapy and immunotherapy for colorectal cancer and radiotherapy for soft tissue sarcoma [62]. However, studies have shown to the contrary that mice fed a diet deficient in carcinogenic methionine and choline develop significant liver fibrosis due to HSC activation and collagen deposition. In addition, most HCC cells undergo cell cycle arrest and severe DNA damage responses after acute methionine deprivation [63]. MAT2A is a rate-limiting enzyme in the methionine cycle that catalyzes the synthesis of SAM from methionine and adenosine triphosphate (ATP). Blocking MAT2A-mediated methionine catabolism leads to the senescence of HCC cells. Combined treatment with MAT2A and GSK3 inhibitors can inhibit the growth of HCC cells both in vitro and in vivo [64]. A high-folate diet promotes the development of HCC in a mouse model induced by a high-fat diet by increasing the expression of MATIIα, while a folate-free diet reduces the expression of MATIIα and hampers HFD-induced HCC development. In addition, MATIIα deletion significantly reduces the levels of folate and multiple intermediate metabolites involved in 1C metabolism. Therefore, folate mediates methionine and 1C metabolism, and promotes HCC development by regulating MATIIα [64]. This finding also suggests that dietary restriction of methionine alone is not effective in hindering the growth of liver tumors.

#### 3.5.3. Folate Mediates DNA Methylation in HCC

Abnormal DNA methylation is closely related to the occurrence and development of HCC. Compared with those in normal liver tissues, 54 CpG islands in 44 genes were hypermethylated, and in these genes, the methylation level of EYA4 was negatively correlated with disease-free survival and overall survival. These results suggest that abnormal hypermethylation of EYA4 may promote HCC progression [65]. The degree of methylation in HCC tissues is greater than that in neighboring non-tumor tissues, and there is a significant correlation between the hypermethylation of the tumor suppressor gene PDCD4 and its down-regulation in cancer tissues [66]. Abnormal methylation of the RASSF1, GSTP1, p14, CDH1, APC, RUNX3, SOCS1, p15, MGMT, SFRP1, WIF1, PRDM2, DAPK1, RARβ, hMLH1, p73, DLC1, p53, SPINT2, OPCML, and WT1 genes in the tumor tissues of HCC patients can be used as a marker to predict the incidence and survival rate of HCC patients [67]. In addition, a study of the methylation of the ADRA1A promoter region in 160 HCC patients showed that the methylation level of the ADRA1A promoter region in the tumor tissues of HCC patients was significantly greater than that in normal tissues, and DNMT inhibitors increased ADRA1A expression in HCC cell lines. These results suggest that ADRA1A gene hypermethylation may be involved in the development of HCC [68]. More than 50% of HCC patients are diagnosed with multifocal hepatocellular carcinoma (mHCC), which has a poor prognosis. mHCC exhibits more complex intra-tumor heterogeneity (ITH) and clonal evolution processes. Consistent with HCC, mHCC also exhibited a high degree of methylation heterogeneity between lesions and patients. Overall hypomethylation has been observed in mHCC lesions compared to paired normal liver tissue. Abnormal DNA methylation of genes may play an important role in the early tumor progression of mHCC [69]. Cancer stem cells (CSCs) promote the occurrence, development, and recurrence of tumors. During cell division, the ubiquitin-like protein UHRF1 with PHD and ring finger domain 1 recruits DNMT1 to a hemimethylated DNA site and maintains DNA methylation, thus allowing daughter cells to inherit DNA methylation patterns. Knockout of UHRF1 in hepatocytes may alleviate DEN/CCL4-induced HCC by regulating the self-renewal and differentiation of hepatic CSCs. Abnormal DNA methylation promotes the occurrence and development of HCC, and folate supplementation promotes the down-regulation of tumor-suppressor gene (TSG) expression in rat models of DEN-induced liver cancer [70]. Therefore, the regulation of DNA methylation by folate may be a major regulatory mechanism in HCC development.

#### 3.5.4. Role of Folate Receptors in HCC

FRs are widely expressed in HCC cells. Treatment with HGF or TGF-β1 can increase the expression of the glycosyltransferase FUT8 and up-regulate the core fucosylation of *N*-glycans on FR FOLR1, thus enhancing folate uptake, and eventually promoting epithelial–mesenchymal transition (EMT) [71], which is the key process of cancer metastasis.

Since FR-α is overexpressed in a variety of cancers, folate has been widely utilized to facilitate the targeted delivery of nanomedicine. For folate-functionalized nanomedical drugs, folate functionalization does not enhance the distribution of liposomes in FR-α overexpressing tumors compared to that of normal liposomes, but leads to increased liposomal capture by macrophages in tumors, the liver, and the spleen. In addition, folate-functionalized polymer nanoparticles are also susceptible to natural IgM absorption, further enhancing the targeting and efficacy of drugs [72]. The introduction of PEG and folate targeting through intra-arterial approaches is also an effective strategy for targeted drug delivery in HCC therapy. PEG has been used to enhance the water solubility of the vector and to increase specific uptake for the interaction of the folate ligand with a variety of FRs [73]. The novel folate-functionalized (doxorubicin, DOX) DOX@ZIF-8 nanoparticles (DOX@ZIF-8-FA) used as a drug delivery system for liver cancer showed increased drug loading and improved sustained drug delivery performance. In HepG2 cells, it has been shown to have greater antitumor efficacy [74]. Through the specific distribution of FRs in HCC cells, the specific binding of folate to FRs can significantly improve the targeting of liver cancer therapy.

#### 3.5.5. Other Mechanisms

Folate exerts anti-tumor activity by inhibiting early angiogenesis in rats, which is related to inhibiting the occurrence and development of HCC [75]. In addition, mitochondrial DNA (mtDNA) deletion and low folate status are carcinogenic features associated with HCC susceptibility. Folate status and liver injury are important factors for mtDNA loss in lymphocytes. In the serum and lymphocytes of 90 HCC patients and 90 healthy controls without cancer, it was found that the serum folate level and lymphocyte folate level of HCC patients were lower, and the cumulative frequency of lymphocyte mtDNA deletion was greater [76]. Both genetic instability of mtDNA and decreased folate levels increase the risk of HCC.

### 3.6. Therapeutic Manipulation of Folate for Liver Disease Treatment and Management

Folate plays an important role in the normal physiological processes in the body. As the main organ of folate storage and metabolism, liver function affects the absorption and metabolism of folate. In addition, folate regulates fatty acid metabolism, the inflammatory response and autophagy, as well as mediating Hcy production. Folate levels also affect liver function. Reduced serum folate levels are found in different liver diseases due to impaired liver function, resulting in malabsorption of folate or insufficient intake of folate. For patients with NAFLD, appropriate folate supplementation may be necessary. Folate supplementation has a protective effect on rats with high fructose intake, which may involve activation of LKB1/AMPK/ACC and increased SAM. This, in turn, inhibits hepatic lipogenesis and ameliorates hepatic steatosis [77]. One randomized controlled trial revealed no significant changes in serum liver enzyme levels, the degree of hepatic steatosis, insulin resistance, or lipid status in patients with NAFLD after 8 weeks of continuous folic acid supplementation (1 mg/day). However, it can inhibit the increase in Hcy. Therefore, the investigators proposed to further study the use of folate of longer duration and at different doses in patients with NAFLD [78]. The study revealed that folic acid treatment was significantly associated with improvements in disease parameters. Folate significantly reduced the expression of the pro-inflammatory cytokines TNF-α, CXCL8, and LC3B. In addition, folate supplementation led to an increase in IL-22 levels in a dose-dependent manner. Folate delays the progression of liver diseases by regulating pro-inflammatory cytokines and autophagy [79]. In addition, the hepatoprotective effects of folic acid on NAFLD may be partly attributed to its anti-inflammatory properties. Folic acid supplementation attenuates hepatic lipid accumulation and inflammatory foci aggregation induced by a high-fat diet. This is associated with NF-κB activation and a significant decrease in inflammatory cytokine expression [79]. Autophagy is an important physiological process for maintaining homeostasis and plays an important role in many liver diseases. Homocysteination and ubiquitination of the autophagy/lysosomal fusion protein syntaxin17 (STX17) lead to autophagic arrest during the development of NASH. VitB12 and folate supplementation restore STX17 expression and autophagy, thereby improving the inflammatory response and fibrosis in NASH [46].

Ethanol-induced oxidative stress and mitochondrial dysfunction are the core pathogenic mechanisms of ALD. Mitophagy is an adaptive quality control mechanism that clears dysfunctional mitochondria and prevents liver injury caused by ALD. Folate reduces alcohol-induced liver injury and oxidative stress. In addition, folate improves mitochondrial function and inhibits ethanol-induced mitophagy by decreasing the expression of PINK1-Parkin and Drp1 expression. This, in turn, inhibits the release of mitochondrial cytochrome C into the cytoplasm, thereby preventing apoptosis [80]. Interestingly, pretreatment of hepatocytes with folate also ameliorates Hcy-induced oxidative damage, mitochondrial division, and mitophagy. Folate has a beneficial effect on the remodeling of mitophagy by scavenging ROS and promoting Hcy metabolism [40]. In addition, folate inhibits ethanol-induced increase in serum triglyceride (TG), total cholesterol (TC) and low-density lipoprotein (LDL), reduces hepatic fat accumulation, and maintains alanine aminotransferase (ALT) at normal levels. Ethanol disrupts liver’s immune homeostasis in the liver, while folate limits ethanol-induced inflammatory damage by increasing the proportion of Treg cells in the liver. Folate supplementation attenuates the ethanol-induced Th3/Treg imbalance by altering the methylation pattern of the Foxp3 promoter [80]. Therefore, folate can be used as a potential treatment for ALD.

The benefits of folate have also been found in viral hepatitis. Supplementation with certain vitamins during pregnancy can affect a baby’s HBsAb levels by increasing cytokine levels. IL-4 mediates the beneficial effects of maternal folate supplementation on HBsAb levels in infants [81]. Folate supplementation in pregnant hepatitis B surface antigen (HBsAg)-positive women promotes the up-regulation of IL-4, which in turn leads to increased anti-HBs levels in infants 11 to 13 months of age [82]. In addition, FRs can also be used as specific therapeutic targets for tumors. By screening patients with colorectal cancer using FR-labeled circulating tumor cells (FR + CTCs) and the metastasis-related marker HSP90, researchers found that colorectal cancer patients with high expression of FR + CTCs and HSP90 were at risk of liver metastasis and had a poorer prognosis [83]. These results indicate that FRs, as tumor-specific markers, can improve the early screening and diagnosis of patients with colorectal cancer. FR-positive tumors are highly specific to folate-PET tracers, and may be regarded as a potential test for the diagnosis and classification of patients with FR-high-expression tumors [84,85]. FRs have a high affinity for folate and its derivatives, and based on this characteristic, imaging agents and therapeutic drugs can be conjugated with folate to target tumor cells, for tumor imaging, such as single photon emission computed tomography (SPECT), nuclear magnetic resonance imaging (NMRI), fluorescence imaging, and tumor treatment, including chemotherapy, isotope therapy, immunotherapy, antisense nucleotide therapy, and gene therapy.

## 4. Conclusions

Abnormal folate levels are associated with the occurrence and development of a variety of liver diseases, and serum folate levels are negatively correlated with liver diseases such as NAFLD, ALD, NASH, liver fibrosis, and HCC. Therefore, folate supplementation is highly important for the treatment of liver diseases. However, excessive folate may lead to a sharp decrease in liver fat and promote the rapid development of HCC, which suggests that patients with liver disease need to be cautious about supplementation with folate. Studies have indicated that folate deficiency is just as harmful as overdose. Folate deficiency promotes the development of liver disease through mechanisms that affect methionine metabolism, 1C metabolism, and epigenetic regulation of genes involved in liver injury. Folate deficiency also leads to increased secretion of pro-inflammatory factors in the liver, impairing hepatic lipid metabolism, and leading to HHcy, hepatic fat accumulation, and fibrosis. Excessive folate can mask VitB12 deficiency, promote the development of HCC, and increase the risk of cancer. Therefore, folate supplementation should be balanced. The recommended intake of folate for the average adult is 400 μg per day. However, because of the fact that the level of folate in patients with liver disease further decreases with the development of the disease, there is no certain standard for the amount of folate supplementation in people with liver disease, indicating that further study is necessary.
